# Dysphagia Days as an Assessment of Clinical Treatment Outcome in Eosinophilic Esophagitis

**DOI:** 10.14309/ajg.0000000000002094

**Published:** 2022-12-20

**Authors:** Ikuo Hirano, Marc E. Rothenberg, Sandra Zhang, Claudia de Oliveira, Christina M. Charriez, Karin S. Coyne, Elizabeth Dansie Bacci, Evan S. Dellon

**Affiliations:** 1 Northwestern University Feinberg School of Medicine, Chicago, Illinois, USA;; 2 Division of Allergy and Immunology, Department of Pediatrics, Cincinnati Children's Hospital Medical Center, Cincinnati, Ohio, USA;; 3 University of Cincinnati College of Medicine, Cincinnati, Ohio, USA;; 4 Bristol Myers Squibb, Princeton, New Jersey, USA;; 5 Evidera, Bethesda, Maryland, USA;; 6 Evidera, Seattle, Washington, USA;; 7 University of North Carolina School of Medicine at Chapel Hill, Chapel Hill, North Carolina, USA.

## Abstract

The aim of this study was to evaluate Dysphagia Days as a measure of symptom improvement in patients with eosinophilic esophagitis from the HEROES study.

**METHODS:**

Dysphagia Days, defined as a yes answer to the following question: During any meal today, did food go down slowly or get stuck in your throat or chest? was assessed for cendakimab vs placebo.

**RESULTS:**

A statistically significant reduction in the mean number of Dysphagia Days experienced was observed with cendakimab 360 mg vs placebo at week 16 (−4.67 vs −1.83; *P* = 0.0115); an even greater improvement was observed in steroid-refractory patients vs placebo (−4.48 vs −0.04; *P* = 0.0079).

**DISCUSSION:**

Dysphagia Days represents a relevant clinical end point to capture dysphagia-related symptoms.

## INTRODUCTION

Finding better treatments of eosinophilic esophagitis (EoE) is an area of active research, but standardization of end points measuring EoE symptoms is lacking. Although the Dysphagia Symptom Questionnaire, EoE Activity Index (EEsAI), and Pediatric EoE Symptom Severity module assess frequency and intensity of dysphagia episodes and have been validated as clinical end point measures for patients with EoE, questions remain about their optimal use (1–3).

Interleukin-13 (IL-13) is implicated in the pathogenesis of EoE (4). Cendakimab is a selective, high-affinity, humanized immunoglobulin G1 monoclonal antibody that recognizes wild-type and variant human IL-13, blocking IL-13 binding to the α1 and α2 subunits of the IL-13 receptors (IL-13Rα1 and IL-13Rα2) with high potency (5). In the randomized, double-blinded, placebo-controlled phase 2 HEROES study (see Supplementary Figure 1, http://links.lww.com/AJG/C794) (6), cendakimab significantly improved histopathologic and endoscopic aspects of disease activity. The mean change in Daily Symptom Diary (DSD) composite score (a different metric than the Dysphagia Symptom Questionnaire), measured over the prior 14 days, resulted in a trend-level improvement with cendakimab 360 mg (*P* = 0.0733) at week 16, despite not being powered to demonstrate clinical symptom improvement. A stronger trend was noted at week 16 in the steroid-refractory group treated with cendakimab 360 mg (*P* = 0.0547). In addition, a trend toward greater reduction in the EEsAI was also observed with cendakimab 360 mg (*P* = 0.1103) in the overall population (6). Cendakimab was well-tolerated; the 360 mg dose was brought forward for evaluation in phase 3 (6).

Given the lack of significant DSD composite score findings, a *post hoc* analysis was conducted to evaluate a potentially new clinical end point termed Dysphagia Days to assess symptom improvement during EoE treatment. Dysphagia Days was chosen based on interviews conducted in a separate validation study where “trouble swallowing,” “food going down slowly,” and “food getting stuck in the throat” were the most relevant symptoms associated with EoE.

## METHODS

Full details of the HEROES study have been reported (6–8). For this *post hoc* analysis, a Dysphagia Day was defined as a yes answer to the following question derived from the DSD: During any meal today, did food go down slowly or get stuck in your throat or chest?

Comparisons of Dysphagia Days between cendakimab and placebo in the intent-to-treat (ITT) population were based on an analysis of covariance model with treatment group, steroid-refractory status, and baseline Dysphagia Days as covariates. Comparisons of cendakimab vs placebo by steroid-refractory status were based on an analysis of covariance model with treatment group, actual steroid-refractory status, and baseline Dysphagia Days as covariates, with treatment group by actual steroid-refractory status as an interaction term. Steroid refractory was defined as failure of an adequate trial of topical steroids to result in meaningful symptom reduction based on investigator assessment (6).

## RESULTS

### Patients

We analyzed 99 ITT population patients, of whom 88 provided change from baseline data for calculating Dysphagia Days (placebo, n = 31; cendakimab 180 mg, n = 28; cendakimab 360 mg, n = 29). Of these, 39 patients were refractory to steroids (placebo, n = 14; cendakimab 180 mg, n = 12; cendakimab 360 mg, n = 13; Table 1).

**Table 1.**
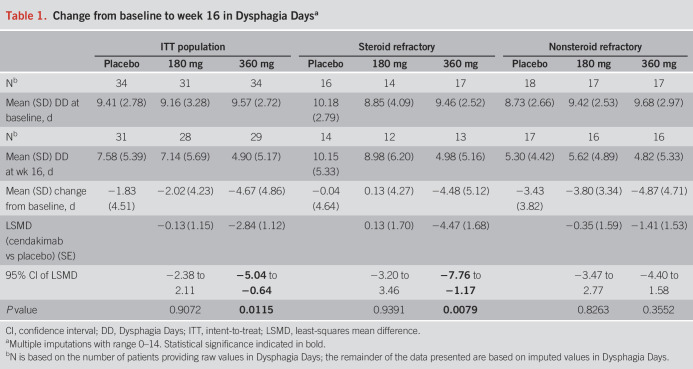
Change from baseline to week 16 in Dysphagia Days^a^

### Dysphagia Days

**Figure 1.**
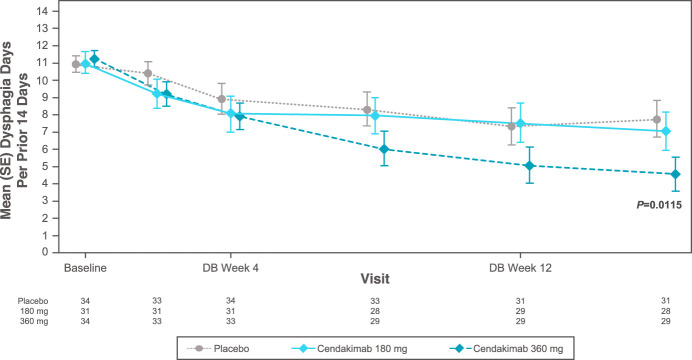
Mean (SE) Dysphagia Days in the double-blinded treatment period (ITT). DB, double-blind; ITT, intent-to-treat.

**Figure 2.**
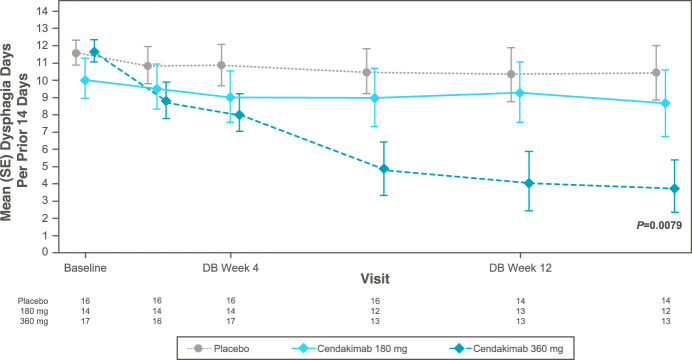
Mean (SE) Dysphagia Days in the double-blinded treatment period (steroid-refractory subgroup). Of the randomized ITT patients, 46.5% were steroid-refractory. DB, double-blind; ITT, intent-to-treat.

At baseline, mean Dysphagia Days experienced over the previous 14 days ranged from 9.16 to 9.57 days in the ITT, 8.85 to 10.18 days in steroid-refractory, and 8.73 to 9.68 days in nonsteroid-refractory populations. At week 16, cendakimab 360 mg weekly significantly reduced the mean number of Dysphagia Days experienced in the overall ITT population vs placebo (−4.67 vs −1.83; least-squares mean difference [SE] −2.84 [1.12]; 95% confidence interval −5.04 to −0.64; *P* = 0.0115; Table 1; Figure 1). A greater reduction in Dysphagia Days occurred with cendakimab 360 mg treatment in the steroid-refractory population vs placebo at week 16 (−4.48 vs −0.04; least-squares mean difference [SE] −4.47 [1.68]; 95% confidence interval −7.76 to −1.17; *P* = 0.0079; Table 1; Figure 2), but no significant differences were observed between cendakimab and placebo in nonsteroid-refractory patients (Table 1). In a separate *post hoc* analysis, Spearman correlation coefficients indicated a strong correlation between Dysphagia Days at day 1 and week 16 and EEsAI (0.61 and 0.83, respectively, both *P* < 0.001), with a higher correlation between Dysphagia Days and DSD composite score (0.91 and 0.96, respectively, both *P* < 0.001) at those time points.

## DISCUSSION

In this analysis, Dysphagia Days was chosen as an end point of interest based on patient interviews in which dysphagia was reported as the hallmark symptom of EoE. Correlation between histologic and symptomatic improvement in EoE is imperfect, however, as noted by the lack of significant improvements in Dysphagia Days with cendakimab 180 mg weekly (Table 1), despite the significant decrease in eosinophil counts observed (6). In this analysis, the lack of a significant reduction in clinical symptoms in the nonsteroid-refractory subgroup may be related to the higher placebo effect observed, which may have blunted the improvement observed with cendakimab. Despite this, reduction in Dysphagia Days provides a clear, objective, and meaningful measure of symptom improvement without interpreting a symptom score. In parallel to this analysis, the Dysphagia Days assessment was further modified and is being used as a coprimary end point in a phase 3 study (NCT04753697) investigating cendakimab in adults and adolescents with EoE, complying with United States Food and Drug Administration guidance for investigation of new drugs for EoE (9). The proportion with eosinophilic histologic response (i.e., peak esophageal eosinophil count ≤6 per high-power field) is the other coprimary end point. Given the simplicity of Dysphagia Days as a patient-reported outcome—targeted to the most common and critical symptom experienced by patients with EoE—and the ease of interpretation from the perspective of the patient and providers, Dysphagia Days has the potential for broader utilization in clinical and community settings for the monitoring of EoE symptoms and could be used as an end point in other diseases where dysphagia is a symptom (e.g., Schatzki rings).

This analysis reveals that change from baseline at week 16 in Dysphagia Days was significant for cendakimab 360 mg vs placebo in both the ITT and steroid-refractory populations. Dysphagia Days may provide a more responsive and clinically relevant end point for the assessment of EoE symptom improvement compared with the DSD composite score. As such, Dysphagia Days is being investigated as a coprimary end point in an ongoing phase 3 trial evaluating cendakimab for the treatment of EoE.

## CONFLICTS OF INTEREST

**Guarantor of the article:** Ikuo Hirano, MD.

**Specific author contributors:** Conceptualization: E.S.D. Data curation: I.H. and S.Z. Investigation: I.H. and E.S.D. Methodology: E.B., E.S.D., S.Z., C.M.C., and K.S.C. Project administration: I.H. Supervision: I.H. Writing–original draft: I.H., S.Z., C.D.O., and C.M.C. Writing–review and editing: All authors.

**Financial support:** This study was supported by Bristol Myers Squibb. Writing and editorial assistance were provided by Claire Jarvis, PhD, and John Simmons, MD, of Peloton Advantage, an OPEN Health company, funded by Bristol Myers Squibb.

**Potential competing interests:** I.H. has served as a consultant for Ellodi/Adare, Allakos, Amgen, Arena, AstraZeneca, Celgene/Receptos/Bristol Myers Squibb, Esocap, Gossamer Bio, Nextsone, Parexel/Calyx, Regeneron/Sanofi, and Shire/Takeda and has received grant/research support from Ellodi/Adare, Allakos, Celgene/Receptos/Bristol Myers Squibb, Sanofi/Regeneron, and Shire/Takeda. M.E.R. has served as a consultant to Pulm One, Spoon Guru, ClostraBio, Serpin Pharm, Allakos, Celldex, Nextstone One, Bristol Myers Squibb, AstraZeneca, Ellodi Pharma, GlaxoSmithKline, Regeneron/Sanofi, Revolo Biotherapeutics, and Guidepoint; has an equity interest in Pulm One, Spoon Guru, ClostraBio, Serpin Pharm, Allakos, Celldex, and Nextstone One; and receives royalties from reslizumab (Teva Pharmaceuticals), PEESSv2 (Mapi Research Trust), and UpToDate. M.E.R. is an inventor of patents owned by Cincinnati Children's Hospital. S.Z, C.d.O., and C.M.C. are employees of Bristol Myers Squibb. K.S.C. and E.D.B. are employees of Evidera. E.S.D. has served as a consultant for Abbott, AbbVie, Adare/Ellodi, Aimmune, Allakos, Amgen, Arena, AstraZeneca, Avir, Biorasi, Calypso, Celgene/Receptos/Bristol Myers Squibb, Celldex, Eli Lilly, EsoCap, GSK, Gossamer Bio, Landos, Morphic, Parexel/Calyx, Regeneron, Robarts/Alimentiv, Salix, Sanofi, and Shire/Takeda; has received grant/research support from Adare/Ellodi, Allakos, AstraZeneca, GSK, Meritage, Miraca, Nutricia, Celgene/Receptos/Bristol Myers Squibb, Regeneron, and Shire/Takeda; and has received educational grants from Allakos, Banner, and Holoclara.

## Supplementary Material

SUPPLEMENTARY MATERIAL
