# Supplementary figures and images for: Dysphagia Days as an Assessment of Clinical Treatment Outcome in Eosinophilic Esophagitis
Source: Am J Gastroenterol. 2022 Dec 20;118(4):744–7. doi: 10.14309/ajg.0000000000002094 (PMC10045973; doi:10.14309/ajg.0000000000002094)

**Supplemental Figure 1.** HEROES Study Design. SC, subcutaneously.


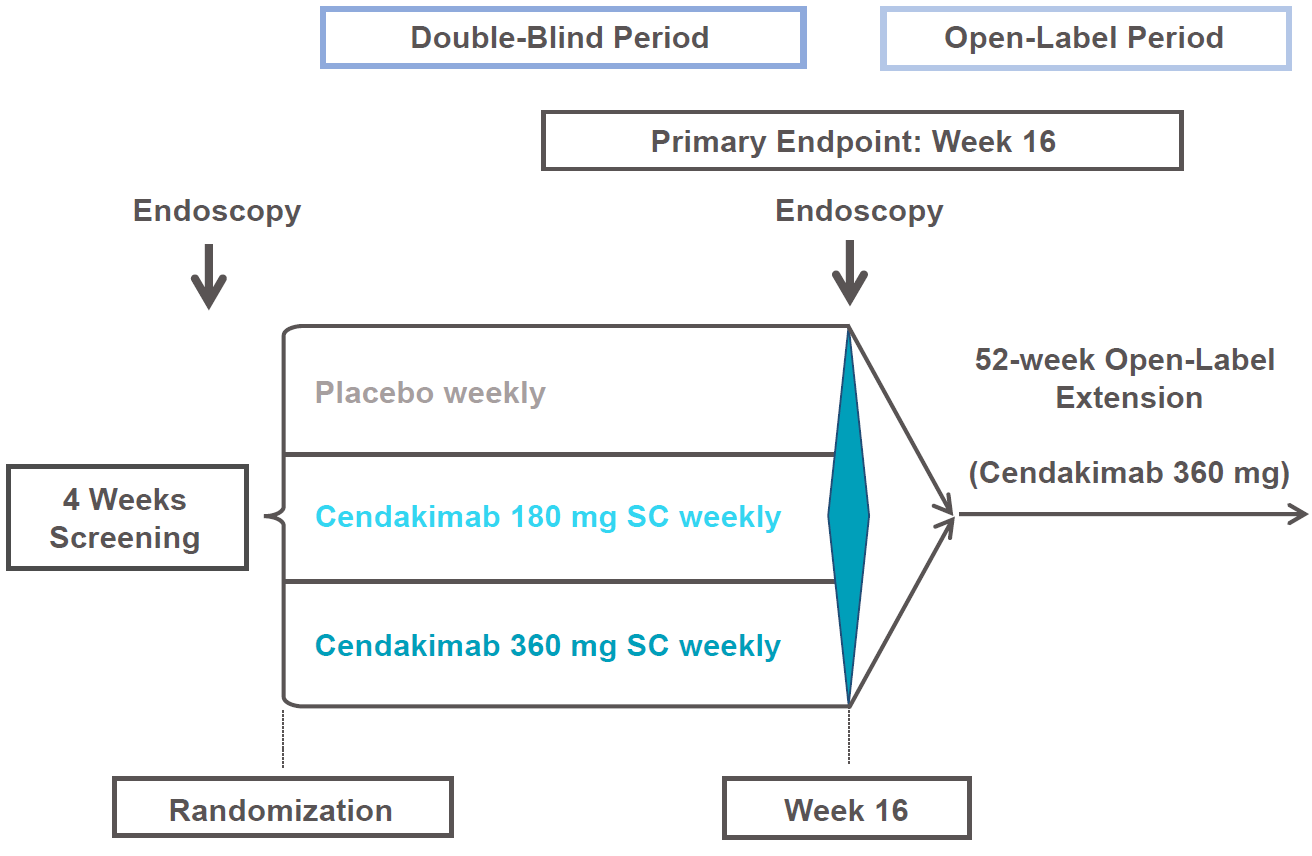

Supplement: SUPPLEMENTARY MATERIAL [file acg-118-744-s001.docx]
